# iQuant2: Software for Rapid and Quantitative Imaging Using Laser Ablation-ICP Mass Spectrometry

**DOI:** 10.5702/massspectrometry.A0065

**Published:** 2018-03-01

**Authors:** Toshihiro Suzuki, Shuhei Sakata, Yoshiki Makino, Hideyuki Obayashi, Seiya Ohara, Kentaro Hattori, Takafumi Hirata

**Affiliations:** 1 Department of Earth and Planetary Sciences, Tokyo Institute of Technology, 2–12–1 Ookayama, Meguro-ku, Tokyo 152–8551, Japan; 2 Division of Earth and Planetary Sciences, Kyoto University, Kitashirakawa Oiwakecho, Kyoto 606–8502, Japan

**Keywords:** laser ablation, ICP mass spectrometry, imaging, software

## Abstract

We report on the development of a software program named iQuant2 which creates visual images from two-dimensional signal intensity data obtained by a laser ablation-ICP-mass spectrometry (LA-ICPMS) technique. Time-resolved signal intensity profiles can be converted to position resolved signal intensity data based on the rastering rate (μm s^−1^) of the laser ablation. Background signal intensities obtained without laser ablation (gas blank) are used as the background, and all of the blank-subtracted intensity data can be used for the imaging analysis. With this software, deformation of the created image can be corrected visually on a PC screen. The line profile analysis between the user-selected points can be observed using the iQuant2 software. To accomplish this, data points on the profile line were automatically calculated based on the interpolation between the analysis points. The resulting imaging data can be exported and stored as JPEG, BMP or PNG formats for further processing. Moreover, a semi-quantitative analysis can be made based on the coupling of the external correction of the RSF (relative sensitivity factor) using NIST SRM610 with normalization of the corrected signal intensity data being 100%. The calculated abundance data for major elements are in reasonable agreement with the values obtained by electron probe micro analyzer (EPMA). With the software developed in this study, both the rapid imaging and semi-quantitative determinations can be made.

## INTRODUCTION

The laser ablation sample introduction technique combined with ICP-mass spectrometry (LA-ICPMS) has been widely used for the rapid, sensitive elemental and isotopic analyses in various solid materials such as minerals, polymers, semiconductors, or steels.^1–6)^ In the LA-ICPMS technique, operational conditions for both the sampling (laser ablation) and ionization can be optimized separately. This is very important in terms of minimizing systematic errors caused by non-mass spectrometric interference (*e.g.*, matrix effects).

One of the recent applications of the LA-ICPMS technique is the imaging analysis of elements or isotopes. With elemental imaging analysis, several unique features, such as the scale or magnitude of sample heterogeneity or secondary movement or the diffusion properties of elements can be evaluated.

A variety of imaging software programs have been already developed and are used to create elemental images from ICPMS measurements, and in many cases, they require a strict data file format in order to satisfactorily construct images. However, such an imaging analysis requires a very long measurement time and it is always possible that such measurements can be interrupted. In such cases, data files that are automatically outputted from ICPMS often cannot completely fulfill the required format. This results in the need to deal with an enormous amount of numerical values in the process of rearranging the files into the requested data style.

We report herein on the development of a new software program named iQuant2, for the imaging of major- to trace-elements based on a laser rastering approach in the LA-ICPMS technique. This software provides image shape correction functions that can dramatically reduce the pre-loading work on data files which do not satisfy the required data style. This software is also designed to provide simple and easy handling for users; most of the operations can be carried out graphically by a mouse click, wheel scrolling or drag-and-drop. Isotope ratio image, correlation analysis, and semi-quantitative analysis can also be achieved by iQuant2.

## METHODS

### LA-ICPMS Analysis

The ICPMS system used in the acquisition of imaging data was an iCAPQc ICPMS (Thermo Fisher Scientific, Bremen, Germany). Two samples (rat brain tissue and silicate) were used as example analytes for the LA-ICPMS imaging in this study. The dwell time used here was 10 ms per isotope, and the number of measured isotopes were 30 in the rat brain sample and 35 in the silicate sample, the resulting time per reading (time step for multi-element monitoring) was 0.3–0.4 s. The laser ablation system used in this study was an NWR 193 ArF Excimer laser (ESI New Wave Research, Portland, USA). The adopted fluence, pit size and repetition rate were 2 J/cm^2^, 75 μm and 5 Hz in the rat brain, and 2.8 J/cm^2^, 10 μm, 20 Hz in the silicate sample, respectively. Laser rastering was achieved by moving the sample stage at a rate of 160 μm s^−1^ with a spacing of 75 μm between the lines in the rat brain, and the values were 10 μm s^−1^ and 11 μm in the case of the silicate sample. The line profile analyses were repeated 195 times to cover the analysis area for the rat brain, while 60 times were used in the case of the silicate sample. During the laser rastering, the signal intensity profile was continuously monitored by the time-resolved analysis mode. The time interval between each rastering line was 30 s, and these intervals are recognized as background measurements in iQuant2. A semi-quantitative analysis was applied to the silicate sample. To define the relative sensitivity factor (RSF) for the analytes, a line profiling analysis under the identical LA-ICPMS conditions with the silicate sample was carried out on the glass standard material (NIST SRM610). The measured signal intensity data were normalized by the concentration of the elements in the NIST SRM610.

### Software Design

The iQuant2 was developed using Windows 7 OS with the Visual Basic 2008 Express Edition, and the resulting software will also run on Windows 8 and 10. To make handling easy and simple, most of the operations of iQuant2 were designed to be carried out by means of a mouse plus a graphical interface; a mouse click, scrolling mouse wheel or drag-and-drop. For example, data loading starts when data file name is dragged-and-dropped from Windows Explorer to Data Input Sub Window of iQuant2. When precise values are required for operations, users can input numerical values using a keyboard.

**Figure 1:**
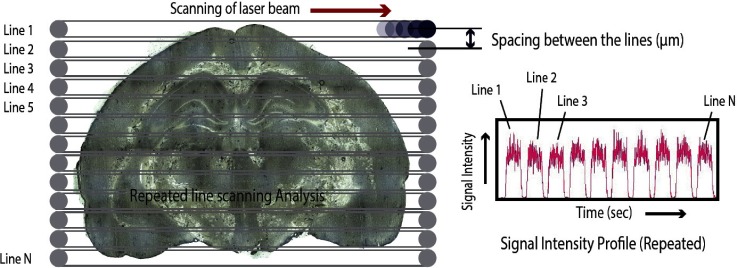
Fig. 1. The concept of the imaging analysis employed in the iQuant2. A series of line analyses are repeated on the sample, and data are accumulated as the time resolved analysis mode of ICPMS.

The basic concept of an imaging analysis employed using iQuant2 is illustrated in Fig. 1. A series of line analyses must be performed in a square area of the sample, and signal intensity data should be obtained by ICPMS as the time resolved analysis (TRA) mode. For data loading, the accumulated data should be output as comma separated values (CSV) formatted files, which is available in most ICPMS instruments. Since the data structure of the outputted CSV file is different in each ICPMS, iQuant2 requires several parameters in order to recognize the structure of data files. iQuant2 will then convert the loaded TRA file to *X*-*Y*-Signal intensity data based on the elapsed time and the rastering rate. An isotope image is created by converting the observed signal intensity to brightness (gray scale) or hue plus brightness (false color) of each pixel.

**Figure 2:**
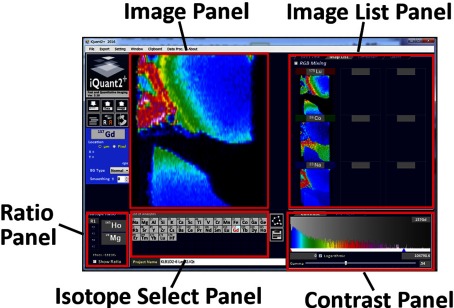
Fig. 2. The main window of iQuant2, which consists of several panels. The false color image is shown on the Image Panel, and the exhibiting isotope can be changed by the Isotope Select Panel. Other isotope images can be listed on the Image List Panel. This panel can be switched to the Bird’s Eye View Panel, the Correlation Panel, and the Quantitative Analysis Panel. Contrast adjustment can be performed by the Contrast Panel, which can be switched to the Line Profile Panel.

An example of the main window of iQuant2 is shown in Fig. 2. The created false color isotope image appears in the middle of the window as the “Image Panel.” Users can easily change the exhibiting isotope image by using the “Isotope Select Panel,” which is located below the Image Panel. The contrast and γ-value of the created image can be optimized by dragging the slider on the “Contrast Panel,” which is located at the bottom right of the window. Isotope images can be compared with each other on the “Image List Panel,” which is placed at the right side of the Image Panel. The images can be listed on this panel by dragging from the Image Panel or the Isotope Select Panel. The RGB mixing function, which will be described in a later section, can be controlled by this panel. By clicking the header list of the Image List Panel, users can switch this panel over to the “Bird’s Eye View Panel,” the “Correlation Panel,” and the “Semi-quantitative Analysis Panel,” which are described below. Users can also switch the Contrast Panel to the “Line Profile Panel” by selecting the headers. Isotope ratios can be calculated and visualized as images by using the “Ratio Panel,” which is located at the bottom left of the window.

## DATA PROCESSING

### Data Loading

**Figure 3:**
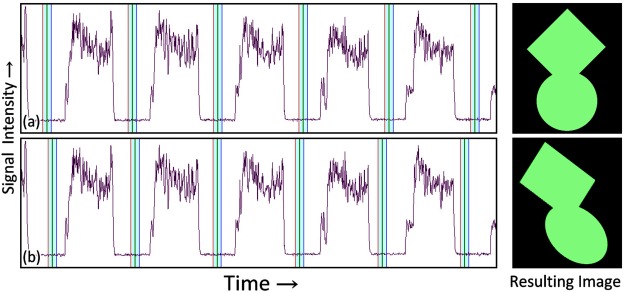
Fig. 3. An example of a signal intensity profile acquired by a raster measurement. The separation points of each line analysis (green vertical lines) and ranges of background (light blue area sandwiched by red and blue line) are semi-automatically defined by iQuant2 and can be visually confirmed by the user. If the errorneous time separation parameter are used (Line Interval Time is slightly short in (b)), the shape of the resulting image will be slanted (b).

The original time resolved data obtained through repeated line profiling analyses is recorded as a sequence of the line intensity profile in data files, and thus, the individual line profile for each analysis line should be separated to construct two-dimensional data. With iQuant2, the overall signal intensity profile can be separated for individual lines based on the time sequence of the measurements. Figure 3 illustrates an example of the measured signal intensity profile. As shown in this figure, the line analysis part and the gas blank part appear in an alternating sequence, and iQuant2 extracts individual line profiles from such data according to the time setting parameters that are input. The separation points, which should be located at around the middle of the gas blank part, are visually indicated by iQuant2, and users can graphically adjust these points. When the separation points are correctly placed, the resulting image exhibits the precise sample shape (Fig. 3(a)). However, the sample shape will be slanted or distorted if the inputted parameter is incorrect (Fig. 3(b)). In such cases, the image can be adjusted by using the Image Shape Correction function of iQuant2, and reloading of data is not necessary. Details of this function are described in the latter section. The gas blank part is also used for estimating background level. If users select the background subtraction option, the background level for each isotope is calculated from the gas blank part and subtracted from the signal intensity data.

### Isotope Image and Bird’s Eye View

**Figure 4:**
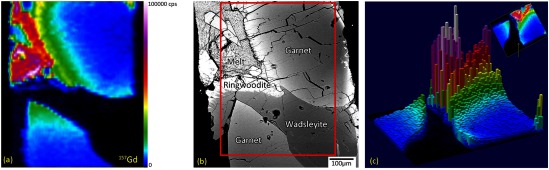
Fig. 4. False color image (a) of ^157^Gd in the silicate sample. The rastered area is shown as a red square in the backscattered electron image of this sample (b). The 3D-object image created from the same data (c) can be shown on the Bird’s Eye View Panel.

The loaded TRA data will be converted into *X*-*Y*-signal intensity data, and iQuant2 creates an isotope image. As an example, the false-color image for ^157^Gd of the silicate sample is shown in Fig. 4(a). A running average can be applied to minimize the contribution of signal spikes, and the resulting image can be immediately shown on the Image Panel. For comparison, a backscattered electron image (BEI) of this sample is shown in Fig. 4(b). This silicate sample is composed of several minerals (garnet, wadsleyite, ringwoodite, some minor phases, and quenched melt), and some minerals show an apparent zoning. Since the brightness of the BEI is controlled by the mean atomic number of the sample, Fig. 4(b) clearly shows that the heavy element content increases from the center to the rim in the garnet and wadsleyite crystals. However, the distribution of each element, especially for trace elements, cannot be determined from the BEI. Figure 4(a) clearly shows that the Gd content in garnet increases in going from the center to the rim, but no obvious change was found in the case of wadsleyite crystals.

Although a false-color image can be very useful in describing the distribution of isotopes, it is difficult to recognize quantitative changes from this image. Moreover, the impression from the false color image can differ from person to person. Hence iQuant2 provides an image of a 3D-object that is created from *X*-*Y*-intensity data. The intensity values at each *X*-*Y* point are converted to the altitude of their locations, and the constructed 3D-object will be shown as a polygon or a wireframe on the Bird’s Eye View Panel. An example of this bird’s eye view image is shown in Fig. 4(c); the intensity of each point is expressed as the height of the tower. These created images can be exported and stored in JPEG, BMP or PNG formats for further processing.

### Image Shape Correction

**Figure 5:**
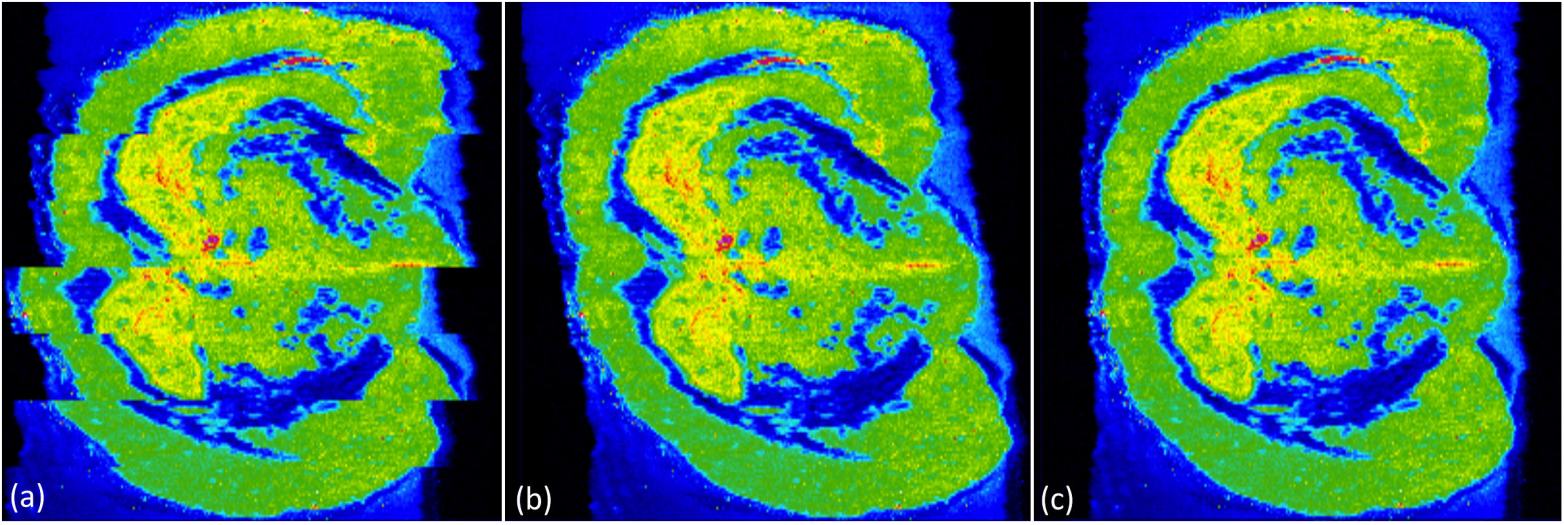
Fig. 5. An example of the image shape correction function of iQuant2. The image directly created from inputted data (a), after the correction of displacements caused by segmentation of data files (b), after the correction of distortion (final image) (c).

If the time setting parameters of data loading do not completely match with the measured conditions, the resulting image can be slanted or distorted. To correct such images, reloading of the data with precise time settings is required when conventional imaging software is being used. Moreover, in some software, the loading data must be a single file, hence users are required to combine the data into a single file if the results were output as separated files. In iQuant2, however, an isotope image can be created from multiple files, and the distortion of the resulting image can be visually adjusted by using the mouse wheel or keyboard keys. An example of such an image shape correction process is shown in Fig. 5. In the case of this example, accumulated TRA data were divided into eight CSV files because of machine time limitations, and moreover, the interval time of the line analysis occasionally fluctuates in the measurements. Therefore, an enormous level of data rearrangement is required if other software is used. Figure 5(a) shows the image just after the data are loaded on iQuant2; image areas created from separate files were horizontally displaced. The horizontal position of each area can be adjusted by rotating the scroll wheel of the PC mouse, and discrepancies between the areas can then be corrected, but the image is still slanted (Fig. 5(b)). The distortion of the image can be also adjusted by the correction function, and horizontal position of each line can also be adjusted. Finally, it is possible to obtain corrected image without reloading or rearrangement of the CSV files (Fig. 5(c)).

### RGB Mixing Image

**Figure 6:**
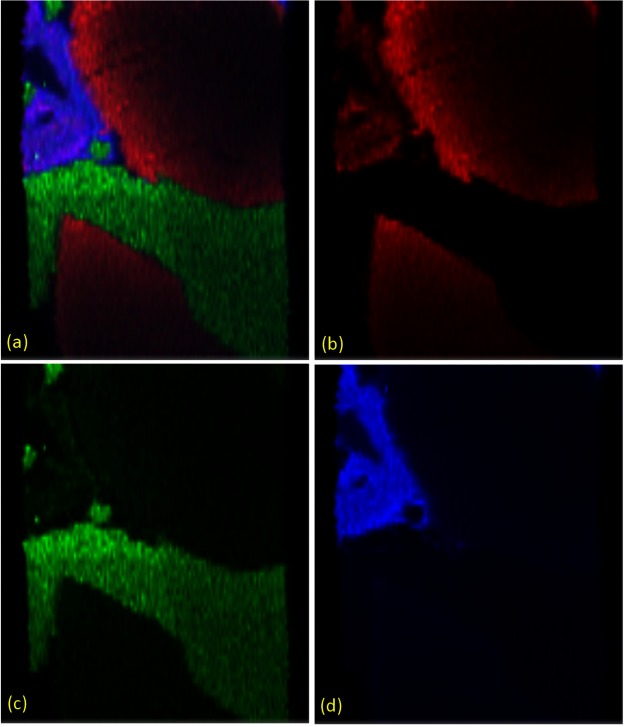
Fig. 6. RGB mixing image of the silicate sample (a). The image of ^175^Lu is assigned to the red channel (b), the green channel is ^59^Co (c), and the blue channel is ^23^Na (d).

With conventional images in which false colors are used, the distribution of only a single isotope can be visualized, which makes comparing the resulting images for different elements a difficult task. To overcome this, an RGB mixing technique was adopted in the iQuant2 software. In the RGB mixing technique, the distribution of three isotopes can be combined to an image using three color components (red, green, and blue). Users can assign the isotope to each color using the Image List Panel. An example of an RGB mixing image for the silicate sample is shown in Fig. 6. In this sample, lutetium (red) is enriched in the garnet and quenched melt, Co (green) is distributed in the wadsleyite and ringwoodite, and the quenched melt contains Na (blue). As the result of color mixing, the garnet is shown as red, wadsleyite and ringwoodite are shown in green, and the quenched residual melt is indicated as magenta (red+blue) in Fig. 6(a). The boundaries between each phase are clearly shown in Fig. 6(a), while they are ambiguous in Figs. 6(b–d).

### Line Profile Analysis

**Figure 7:**
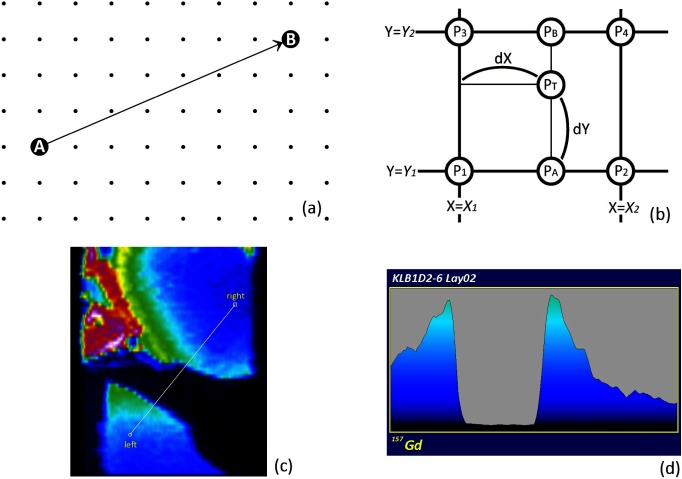
Fig. 7. Imaging data is composed of discrete data points (solid circles in (a)) distributed at equal intervals. When a line profile from point A to B is required, the line scarcely passes over the data points. Hence the values at intermediate points (open squares) must be estimated from nearby measured points to acquire line profile. Signal intensity at point P_T_, which is surrounded by P_1_–P_4_, will be calculated from the observed intensity of P_1_–P_4_ (b) using Equation (4) in the main text. The line profile along the line indicated in (c) was constructed by this procedure and shown in (d). The starting point and the ending point shown in (c) can be selected by using the Image Panel.

With the iQuant2 software, the line profile for the signal intensity between two selected points can be indicated on the Line Profile Panel, and the start point and end point of the line profile can be specified by the mouse on the Image Panel (see Fig. 7). Since imaging data are composed of two-dimensional data arrays obtained by repeated line-profiling analyses, the data points are spatially discrete in the *X*-*Y*-intensity data space (Fig. 7(a)). To draw a line profile, signal intensity values for the points in the intermediate space must be calculated. In the iQuant2 software, the following procedure is employed to calculate the value for the intermediate data points. In Fig. 7(b), P_1_–P_4_ show the location of the measured points, and the intensity observed at these points is expressed as *I*_1_–*I*_4_. P_T_ is the target point which is located at (*X*_1_+d*X*, *Y*_1_+d*Y*). The intensity at point P_A_, whose location is (*X*_1_+d*X*, *Y*_1_), can be calculated by linear interpolation. 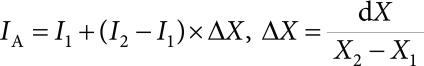
(1)

The value at point P_B_ can be also calculated as 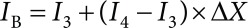
(2)

The value at P_T_ can then be acquired from *I*_A_ and *I*_B_ using the same procedure. 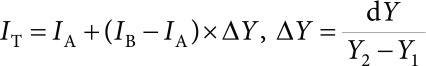
(3)

Thus, using the above equations, *I*_T_ is calculated by the following equation. 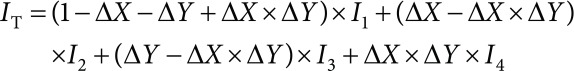
(4)

### Correlation Analysis

**Figure 8:**
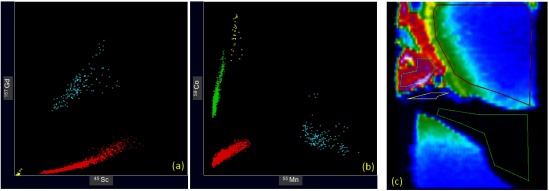
Fig. 8. Correlation between signal intensity(CPS) of ^45^Sc–^157^Gd (a) and ^55^Mn–^59^Co (b) observed in the silicate sample. The selected polygonal areas are shown in (c); red=garnet, green=wadsleyite, yellow=ringwoodite, light blue=quenched residual melt.

The iQuant2 program can also be used to visualize correlations between the signal intensity of isotopes by using the Correlation Panel. Figure 8 illustrates an example of such an analysis, showing the correlation between signal intensity of ^45^Sc–^157^Gd and ^55^Mn–^59^Co in the silicate sample. In iQuant2, users can pick up polygonal areas on the Image Panel (Fig. 8(c)), and the data points included in these areas are shown in different colors on the Correlation Panel. Since minerals show their individual geochemical behaviors, different correlations are found on these figures.

### Semi-Quantitative Analysis

The false color images provide qualitative information regarding the distribution of elements and isotopes. For a quantitative discussion of the diffusion or movements of elements, however, the concentrations of these components in the sample would be highly desirable. To meet such demands, the iQuant2 program can provide rough estimations of elemental concentrations by the Semi-Quantitative Analysis functions. In this function, we employed a simple quantitative calculation process which is widely used in LA-ICPMS analysis.^7,8)^ The procedures are as follows.

In a laser ablation measurement, the number of isotopes generated by the ablation of the sample can be expressed as 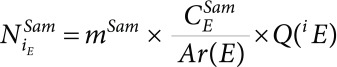
(5)where *N_^i^E_^Sam^* is the number of isotope *i* of element *E*, *m^Sam^* is the mass of the ablated sample, *C_E_^Sam^* is the concentration of *E* in the sample, *Ar*(*E*) is the atomic weight of element *E* and *Q*(*^i^E*) is isotope abundance of *^i^E*. If we assume that the signal intensity of *^i^E* measured by mass spectrometer, *I_^i^E_^Sam^*, is a linear function of *N_^i^E_^Sam^*, the equation is written as 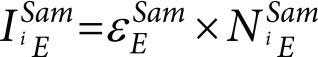
(6)where ε*_E_*^Sam^ is the sensitivity factor of mass spectrometer of element *E*. Then *I_^i^E_^Sam^* can be expressed as 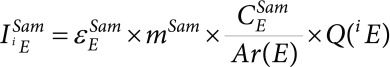
(7)

This equation is also applied to the ablation of the standard. When we calculate the ratio of the signal intensity of the sample and standard, *Ar*(*E*) and *Q*(*^i^E*) in the denominator and numerator are canceled out, and the ratio is written as 
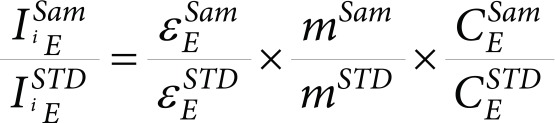
(8)

The concentration of *E* in the sample can then be calculated as 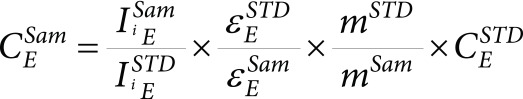
(9)

Since *m^STD^* and *m^Sam^* do not change with element, the ratio of mass and sensitivity factor can be written as constant α if we assume that the ratio (ε*_E_*^STD^/ε*_E_^Sam^*) is constant in all elements, and we can obtain the following equation. 
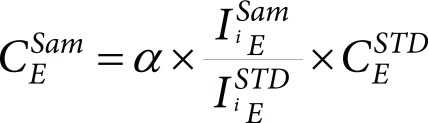
(10)

Since *m^Sam^* will change in every measurement point, α-value must be calculated in each measurement point.

If we have concentration data for an element measured by another analytical method, such as electron probe micro analyzer (EPMA), then α can be calculated. This “internal standardization method” has been adopted in quantitative analysis by LA-ICPMS.^7,8)^ For example, in the case of earth sciences, Si is often used as this “internal standard element,” and α is calculated using the measured *C_Si_^Sam^*. 
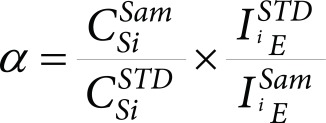
(11)

Thus, we can acquire quantitative data for the sample, but the applicable area of this method is limited to the points where the Si concentration is acquired beforehand.

There is another way to acquire the value of α. Since the summation of all element concentrations must be 1, we can use the following equation if all of the elements present in the sample are measured.^9,10)^

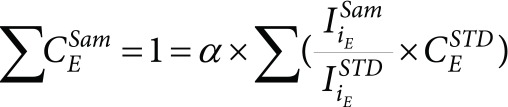
(12)

In this paper, we refer to this procedure as the “100% normalization method.” To conduct this method, it is necessary to measure all of the elements that are present in the sample. In the case of the present sample, the most abundant element in the mineral sample is oxygen which cannot be measured by LA-ICPMS. Therefore, we added the function called the “oxide mode” in the 100% normalization method, which assumes that all measured elements are present in the form of oxides in the sample, and quantitative results are expressed as oxides. Although some elements can have multiple valence states, the valence is fixed in each element. For example, iron is always calculated as FeO. Similar procedures are often employed in EPMA because the precise measurement of oxygen is also not easy. Using above “internal standardization method” or “100% normalization method,” iQuant2 calculates the concentration of elements by a point, an average of a rectangular area, and an average of a polygonal area.

**Figure 9:**
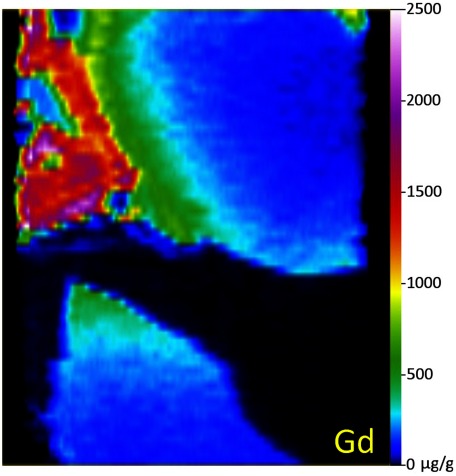
Fig. 9. The False color image of Gd concentration map created by “100% normalization method.” Signal intensity image of this area is shown in Fig. 4(a).

Since the 100% normalization method requires no other quantitative measurements, this procedure can be applied to all measured points and the “signal intensity image” can be converted to a “concentration image.” This is the advantage of the 100% normalization method. The semi-quantitative image of Gd created by this method is shown in Fig. 9.

## DISCUSSION

### Spatial Resolution of Image

With the iQuant2 software, the elemental and isotopic imaging is constructed based on the repeated analysis with the line profiling of signal intensities using rastering the laser beam on the area of the sample of interest. Positions of the data were calculated from the raster speed and the elapsed time. Hence, the spatial resolution can change with various parameters such as the raster speed of the laser, the magnitude of signal tailing (*i.e.*, washout time), time resolution of the data acquisition (*i.e.*, dwell time and number of isotopes that are being monitored). This suggests that the resulting spatial resolution can be different among different instrumental setups, even under identical analytical conditions. Moreover, it should be noted that spatial resolution for two directions (*i.e.*, along the laser rastering direction and the vertical direction against laser rastering) can be basically different. Spatial resolution along the line profiling can be improved by slowing the laser rastering or shortening the sample washout time, whereas the spatial resolution in the vertical direction is basically defined by the size of the ablation pit size. Hence, the resulting spatial resolution can be seriously affected by many analytical conditions and types of instrumentation, and it is not possible for us to make a numerical evaluation on the spatial resolution in this section.

In the case of imaging analysis using the LA-ICPMS, there is a significant time delay between the timing of laser ablation and the timing of the signal detection. This is mainly due to the long delivery time for the laser-induced sample aerosol from the laser ablation cell onto the ICP ion source. The time required for the transport of the sample aerosol would typically be 2–3 s when a sample transport tubing with a length of 2–3 m is used. This time delay can cause large uncertainties in the identification of the positions of the elements. Moreover, it should be noted that laser-induced sample particles can undergo diffusion during their transport. This suggests that the resulting signal intensity profile, even produced by the single laser shot, can be elongated. As a result, signals obtained with multiple laser shots can be mixed when a laser ablation was performed with a shorter time interval than the time scale of the signal extension (*i.e.*, 2 s). Great care must be made to avoid the mixing of the sample aerosols produced by the multiple-laser shots, and thus, the laser repetition rate and laser rastering speed must be carefully optimized to minimize the contribution of sample mixing. The iQuant2 program simply assigns the TRA signal to a series of spot data and create an isotope image. For example, the image shown in Fig. 4 is composed of approximately 3 dots/10 μm in the horizontal direction, and 1 dot/11 μm in the vertical direction. However, it should be noted that this does not mean that the resulting spatial resolution for the horizontal direction was better than that for the vertical direction, because of the contribution of extension and mixing of the sample aerosols through the sample transport process. Moreover, the running-average with ±2 data points was applied in the image of Fig. 4. This procedure can result in the further lowering the resolution of the resulting images along the laser raster direction. Many efforts have been made to minimize the contribution of signal tails, fast washout cells, and sample delivery systems.^11,12)^ With the fast washout system, although a shorter analysis time can be achieved, the number of elements or isotopes are severely restricted due to the limited mass scan rate of the conventional mass spectrometer.

The contribution of sample mixing during sample transport can be minimized by multiple spot analyses, in that the laser beam was no longer rastered. Based on a repeated single laser shot analysis, signal intensity data produced by the single ablation pit would be totally integrated, thus obviating the risk of the mixing of the laser aerosol released from different ablation pits. Based on the multiple-spot protocol, it is possible to accurately identify the positions of elements that are released from the sample. Nevertheless, the time for the measurement becomes longer because the time interval between the analysis spots should be longer than the time for the tailing of the signal washout time. In the case of the present system setup (*e.g.*, the signal washout time was about 2 s), the analysis time for imaging using multiple spot analysis would be about 10 times longer than that achieved by the multiple-line profiling analysis. Despite the obvious success in obtaining better spatial resolution, to take full advantage of the LA-ICPMS technique, we adopted a line-profiling protocol for the imaging simply because of the shorter analysis time. In fact, for larger-sized samples (>10 mm×10 mm), the spatial resolution of about 100 μm would be sufficiently high enough for this discussion.

It should be noted here that the numerical correction for the contribution of signal tails can improve the spatial resolution of the raster imaging.^13)^ The correction technique they used was a simple linear dynamic model based on the mathematical correction of a dead-time lag and a higher-order lag representing aerosol transport through the tubing in the laminar flow regime.^14)^ Although this correction process requires very long calculation times, the spatial resolutions achieved by the laser rastering protocol were almost comparable to that achieved by the multi-spot approach. This suggests that the laser rastering approach is beneficial for both a simple system configuration and multi-element capability of the analysis.

### Uncertainty of Semi-Quantitative Analysis

For precise quantitative analysis, several effects (matrix effect, monoatomic and polyatomic interferences, *etc.*) must be taken into consideration for evaluating the analytical uncertainties of the results. As described in a previous section, we employed simple assumptions for the calculation procedures in semi-quantitative analysis,^7–10)^ and the calculated values can include a certain amount of uncertainties in many cases. As an example of the uncertainty associated with the acquired values, rectangular areas at the central part of the silicate mineral (garnet and wadsleyite) were chosen, and the results obtained by the 100% normalization method are summarized in Table 1. In these calculations, a line scan result of NIST610 was used as the standard. For comparison, data obtained by the EPMA technique are also listed in Table 1. These results indicate that the systematic discrepancy in the present semi-quantitative analysis for major components was generally less than 15%. We therefore conclude that the results are reasonable for use as “semi-quantitative calculations” of an imaging analysis. These uncertainties might be changed when different ablation conditions (scan speed, fluence of laser, *etc.*) are employed.

**Table 1:** Table 1. Comparison of major element contents obtained by EPMA and 100% normarizing method.

Garnet				
	EPMA	LA-ICPMS	*N*=150	
		Avr.	S.D.	(ICPMS/EPMA)−1
SiO_2_	54.36	58.00	3.76	0.067
TiO_2_	0.08	0.04	0.01	−0.468
Al_2_O_3_	5.64	4.88	0.67	−0.134
CaO	2.34	1.65	0.54	−0.296
MgO	33.98	32.01	3.46	−0.058
FeO	2.89	2.62	0.41	−0.094
MnO	0.09	0.09	0.15	−0.021
NiO	0.03	n.m.		
Na_2_O	0.12	0.14	0.02	0.115
Cr_2_O_3_	0.34	0.36	0.05	0.071
Total	99.87	99.79		
Wadsleyite				
	EPMA	LA-ICPMS	*N*=147	
		Avr.	S.D.	(ICPMS/EPMA)−1
SiO_2_	40.91	42.60	3.12	0.041
TiO_2_	0.02	0.01	0.00	−0.524
Al_2_O_3_	0.32	0.27	0.05	−0.154
CaO	0.03	0.05	0.07	0.533
MgO	51.93	50.24	3.25	−0.033
FeO	5.97	6.51	0.80	0.091
MnO	0.06	0.08	0.01	0.206
NiO	0.20	n.m.		
Na_2_O	0.07	0.09	0.02	0.358
Cr_2_O_3_	0.08	0.10	0.01	0.250
Total	99.59	99.94		

S.D.=1σ

## CONCLUSION

We report on the development of a software program for Windows PC named iQuant2, which can analyze two-dimensional data accumulated by raster analysis using LA-ICPMS. This software can provide not only visual data such as isotope images and line profiles but also semi-quantitative analyses. The user interface of iQuant2 is designed so as to permit the operations to be both simple and easy. Coupled with the high sensitivity in LA-ICPMS, this software has the potential to be a powerful tool for a wide range of research fields, such as earth sciences, biochemistry and the material sciences. For further information on the use of the iQuant2 software and related materials, please contact suzuki@eqchem.s.u-tokyo.ac.jp.

## Notes

Mass Spectrom (Tokyo) 2018; 7(1): A0065
